# Association of Maternal History of Spontaneous Abortion and Stillbirth With Risk of Congenital Heart Disease in Offspring of Women With vs Without Type 2 Diabetes

**DOI:** 10.1001/jamanetworkopen.2021.33805

**Published:** 2021-11-10

**Authors:** Honglei Ji, Hong Liang, Yongfu Yu, Ziliang Wang, Wei Yuan, Xu Qian, Ellen Margrethe Mikkelsen, Anne Sofie Dam Laursen, GuangHong Fang, Guoying Huang, Maohua Miao, Jiong Li

**Affiliations:** 1 School of Public Health, NHC Key Laboratory of Reproduction Regulation (Shanghai Institute for Biomedical and Pharmaceutical Technologies), Fudan University, Shanghai, China; 2 Department of Clinical Epidemiology, Aarhus University, Aarhus, Denmark; 3 NHC Key Laboratory of Reproduction Regulation (Shanghai Institute for Biomedical and Pharmaceutical Technologies), Fudan University, Shanghai, China; 4 Department of Biostatistics, School of Public Health, The Key Laboratory of Public Health Safety of Ministry of Education, Fudan University, Shanghai, China; 5 Department of Maternal, Child and Adolescent Health, School of Public Health and Global Health Institute, Fudan University, Shanghai, China; 6 Shanghai Key Laboratory of Birth Defects, Children’s Hospital of Fudan University, Shanghai, China; 7 Ministry of Education–Shanghai Key Laboratory of Children’s Environmental Health, Xinhua Hospital, Shanghai Jiao Tong University School of Medicine, Shanghai, China

## Abstract

**Importance:**

The associations of maternal history of spontaneous abortion (SA) and stillbirth with congenital heart disease (CHD) remain elusive.

**Objective:**

To evaluate the associations of maternal history of pregnancy loss with CHD in offspring and the role of maternal type 2 diabetes.

**Design, Setting, and Participants:**

This population-based cohort study included singleton live offspring born between January 1, 1977, and December 31, 2016, identified through Danish national health registries. Statistical analysis was performed from October 1, 2019, through September 1, 2021.

**Exposures:**

Maternal history of SA, with frequency varying from 1 or 2 to 3 or more episodes, and maternal history of single and multiple stillbirths.

**Main Outcomes and Measures:**

Overall CHD identified by hospital diagnosis. Cox proportional hazard regression was used to estimate the hazard ratio (HR) of CHD. Diabetes was evaluated as a potential confounder and a potential effect modifier.

**Results:**

Among 1 642 534 included offspring (mean [SD] age, 14.11 [8.39] years; 843 265 male [51.35%]), 246 669 (15.02%) were born to mothers with a history of SA and 9750 (0.59%) were born to mothers with a history of stillbirth. The HRs of CHD were 1.16 (95% CI, 1.13-1.20) for offspring with a maternal history of SA and 1.49 (95% CI, 1.32-1.68) for offspring with a maternal history of stillbirth. Significant dose-response associations were observed among offspring with a maternal history of 3 or more episodes of SA (HR, 1.60; 95% CI, 1.39-1.84) and those with maternal history of multiple stillbirths (HR, 2.75; 95% CI, 1.63-4.65). If only inpatient CHD cases were included, the risk of CHD was higher than that found in the main analysis, with HRs of 1.24 (95% CI, 1.19-1.30) for maternal history of SA and 1.78 (95% CI, 1.51-2.11) for maternal history of stillbirth. The observed associations were strengthened by maternal prepregnancy type 2 diabetes (HR for maternal history of SA, 1.65 [95% CI, 1.37-1.97]; HR for maternal history of stillbirth, 1.74 [95% CI, 1.06-2.85]).

**Conclusions and Relevance:**

These findings suggest that offspring born to mothers with a previous SA or stillbirth, especially multiple episodes, or with prepregnancy type 2 diabetes were at a higher risk of being diagnosed with CHD. These findings may help identify women at increased risk in whom detailed fetal heart assessment may be cost-effective and highlight the importance of screening for type 2 diabetes in women of reproductive age.

## Key Points

**Question:**

Is the risk of congenital heart disease associated with a maternal history of pregnancy loss?

**Findings:**

In this cohort study of 1 642 534 offspring born between 1977 and 2016 in Denmark, maternal history of spontaneous abortion (SA) and stillbirth were associated with increased risks of overall congenital heart disease (CHD) in children of 16% and 49%, respectively. The associations were further strengthened by maternal prepregnancy type 2 diabetes.

**Meaning:**

These findings help to identify women at increased risk in whom detailed fetal heart assessment may be cost-effective and highlight the importance of screening for type 2 diabetes in women of reproductive age.

## Teaser

This cohort study investigates the associations of maternal history of spontaneous abortion and stillbirth with congenital heart disease in offspring in Denmark and evaluates whether the association is strengthened by the presence of maternal type 2 diabetes.

## Introduction

Congenital heart disease (CHD) is the most common congenital anomaly, with a global prevalence ranging from 9 to 18 per 1000 live births that resulted in 2.6 million deaths worldwide in 2017.^1,2^ The etiology of CHD is complex, and epidemiological studies have suggested that a genetic or environmental cause can be identified in 20% to 30% of CHD cases.^3^ Several environmental factors have been recognized previously, including maternal smoking, prepregnancy diabetes, thyroid disorder, and prepregnancy obesity.^4,5,6^ Because universal prenatal screening for CHD is not feasible in many countries and regions, leading to a relatively low prenatal detection rate of CHD, it is important to identify prenatal risk factors to determine high-risk groups who merit detailed assessment of fetal heart function.^7,8,9^

Maternal history of pregnancy loss, including spontaneous abortion (SA) and stillbirth, was suggested as a risk factor for CHD in offspring in 2 case-control studies^10,11^ and 1 cross-sectional study.^12^ However, a null association was reported in another case-control study.^13^ The inconsistent findings and less favorable design of these studies make it difficult to draw a solid conclusion. For example, 1 study^10^ reported crude estimates and failed to control the effects of the potential confounders such as maternal age at delivery, while another study^12^ only investigated the association between recurrent SA (defined as 3 or more SAs) and CHD. Spontaneous abortion occurs in 15% of all clinically identified pregnancies,^14^ and stillbirth accounts for 18.4 per 1000 total births in the world and 3.4 per 1000 in developed regions.^15^ The relatively high prevalence of pregnancy loss means that a small increased risk of CHD associated with maternal history of pregnancy loss could have major public health implications. Thus, the determination of maternal history of SA and stillbirth as risk factors for CHD warrants further investigation by well-designed studies.

It has been reported that maternal history of SA and stillbirth may co-occur with maternal chronic diseases, including diabetes.^16,17^ One previous study showed that maternal diabetes was strongly associated with CHD in offspring (relative risk of 4.0).^4^ It has also been suggested that diabetes and pregnancy loss might share common pathophysiology such as autoimmune disorders and subclinical inflammatory processes.^18^ Thus, an interplay of maternal diabetes and pregnancy loss in the association with CHD among offspring may exist. However, the joint association has not yet been investigated.

In this large population-based cohort study, we aimed to investigate the association of maternal history of SA and stillbirth with CHD in offspring. We also investigated the joint association of maternal diabetes and maternal history of SA or stillbirth with CHD.

## Methods

### Study Population

We established a population-based cohort using data from national registries in Denmark. Accurate individual-level linkage of data from national registries was performed using the unique personal identification number assigned to all residents. The study was approved by the Danish Data Protection Agency. By Danish law, no informed consent is required for a register-based study of anonymized data. This report followed the Strengthening the Reporting of Observational Studies in Epidemiology (STROBE) reporting guideline for cohort studies.

From the Danish Civil Registration System, we identified all 2 393 576 singleton live births in Denmark between January 1, 1977, and December 31, 2016. The participants were linked to data from the Danish National Patient Register (DNPR), the Danish National Prescription Database, the Danish Medical Birth Register, the Danish Register of Causes of Death, and the Danish Integrated Database for Labor Market Research.^19^ The DNPR contains inpatient admissions from 1977 onward and outpatient and emergency department data from 1995 onward in Denmark. The Danish National Prescription Database has held information on redeemed prescriptions since 1995. We excluded births in which the mother was younger than 15 years in 1977, when the DNPR was established, to be able to identify a registered SA for all mothers of included children. The final cohort included 1 642 534 singleton live births.

### Exposure

Offspring were categorized as exposed if their mothers had at least 1 SA diagnosis before pregnancy of the index child, as recorded in the DNPR using the *International Classification of Diseases, Eighth Revision* (*ICD-8*), until the end of 1993 and *International Statistical Classification of Diseases and Related Health Problems, Tenth Revision* (*ICD-10*) thereafter (eTable 1 in the Supplement). Women were assumed to have experienced SA twice if they were registered with SA more than once and the interval between the first date of diagnosis and a subsequent date of diagnosis was longer than 90 days. Subsequent SAs were determined in a similar way. Correspondingly, we categorized exposed offspring into 3 groups: those with mothers experiencing SA 1, 2, and 3 or more times.

Offspring born to mothers with records of stillbirth in the Danish Civil Registration System before pregnancy of the index child were also identified as exposed. We divided exposed offspring into maternal history of a single stillbirth and multiple (≥2) stillbirths, depending on the number of stillbirths recorded.

### Maternal Diabetes

We retrieved the diagnosis of diabetes from the DNPR and diabetes-related medication from the Danish National Prescription Database, coded in accordance with the Anatomical Therapeutic Chemical (ATC) classification. We defined diabetes by at least 2 prescriptions of insulin and analogues (ATC code A10A) or drugs other than insulin to lower blood glucose levels (ATC code A10B) for the same woman. We categorized diabetes into 3 subtypes: prepregnancy type 1 diabetes (*ICD-8* code 249; *ICD-10* codes E10 and O24.0; ATC code A10A), prepregnancy type 2 diabetes (*ICD-8* code 250; *ICD-10* codes E11 and O24.1; ATC code A10B), and gestational diabetes in the index pregnancy (*ICD-8* codes 634.74 and Y6449; *ICD-10* codes O24.4 and O24.9). The date of the first occurrence of diabetes in the DNPR or the Danish National Prescription Database was defined as the time of onset. If a mother was diagnosed with multiple types of diabetes, she was classified according to the first diagnosed type.

### Follow-up and Outcome

The outcome of interest was either a diagnosis of CHD identified from the DNPR or death due to CHD identified in the Danish Register of Causes of Death (*ICD-8* codes 746-747 and *ICD-10* codes Q20-Q26, except for *ICD-8* codes 746.7, 747.5-747.9 and *ICD-10* codes Q26.5-Q26.6, which are not specific to CHD). The date of the first diagnosis of CHD or date of death was identified as the diagnostic date of the disease. All offspring were followed up from birth until the onset of CHD, death, emigration, or end of follow-up on December 31, 2016, whichever came first.

### Covariates

The covariates were chosen a priori. Maternal history of CHD and hypothyroidism were identified from the DNPR (eTable 1 in the Supplement). In the main analyses, potential confounders included birth order (1, 2, and ≥3), maternal age at birth (<20, 20-24, 25-29, 30-34, and ≥35 years), maternal cohabitation at delivery (single or cohabitating), maternal educational level (primary and lower secondary, upper secondary or academic degree, and university education at bachelor’s degree level or higher), maternal CHD (yes or no), maternal hypothyroidism (yes or no), and paternal age at birth (<20, 20-24, 25-29, 30-34, and ≥35 years). We also included child sex (boy or girl) and birth year (1977-1991, 1992-1996, 1997-2001, 2002-2006, 2007-2011, and 2012-2016). We used multiple imputation to deal with missing values of the following covariates: child sex (0.02% missed), maternal cohabitation (0.20% missed), maternal educational level (2.62% missed), and paternal age at birth (1.75% missed).

### Statistical Analysis

We used Cox proportional hazard regression to estimate the hazard ratios (HRs) and 95% CI of CHD for exposed compared with unexposed offspring. Furthermore, we examined whether the strength of associations increased with more episodes of SA and stillbirth. A *P* value for trend was obtained by introducing the number of SAs (0, 1, 2, or ≥3) or stillbirths (0, 1, or ≥2) as a continuous variable into the model, with 2-sided *P* < .05 indicating statistical significance. We further restricted the analyses to inpatient cases of CHD to investigate whether the associations were more evident in severe CHD cases. We examined potential effect modification by child sex in sex-stratified analyses.

We evaluated 3 subtypes of maternal diabetes as potential confounders by adjusting for them independently. We then evaluated whether maternal diabetes modified the association between maternal history of SA and CHD in offspring by including an interaction term between maternal diabetes and maternal history of SA. The HRs and 95% CI of CHD were calculated for offspring with only maternal history of SA, with only maternal diabetes, and with both compared with those without maternal history of SA or diabetes. We also presented estimates for maternal history of SA within the strata of maternal diabetes. The potential joint association of maternal diabetes and maternal history of stillbirth with CHD was tested in a similar way.

We performed the following sensitivity analyses to test the robustness of the findings. First, we reran the model after excluding participants with missing values of covariates, including child sex, maternal cohabitation, maternal educational level, or paternal age at birth, to compare with the main result. Second, we performed subgroup analyses among the first live-born children to examine whether our observed association was affected by the sibling aggregation of CHD. Third, we restricted the analyses examining the association of maternal history of pregnancy loss with CHD in offspring and the potential effect modification of maternal diabetes to offspring born after 1995, when outpatient records and emergency department data were included in the DNPR, as well as when the Danish National Prescription Database was established. Fourth, we reran the models among offspring born after 1994, when *ICD-10* was brought into use in Danish registries. Fifth, before January 1, 2004, SA in Denmark was defined as pregnancy loss at or before 28 gestational weeks, whereas a fetal death after that was regarded as a stillbirth. In 2004, this cut point was changed to 22 gestational weeks. To address the inconsistency due to the change of the criterion, we repeated the analysis after reclassifying all fetal deaths from 22 to 28 gestational weeks as SA. Sixth, because data on maternal smoking (yes or no) were available since 1991, we additionally adjusted for maternal smoking in a subset of offspring born after 1991. Seventh, for the same reason, we adjusted for maternal prepregnancy body mass index (calculated as weight in kilograms divided by height in square meters and categorized as <18.5 [underweight], 18.5-24.9 [normal weight], 25.0-29.9 [overweight], and ≥30.0 [obesity]) among offspring born after 2004. Data handling and statistical analyses were performed using SAS, version 9.4 (SAS Institute Inc).

## Results

### Description of the Exposure

Among 1 642 534 included offspring (mean [SD] age, 14.11 [8.39] years; 799 005 [48.65%] female and 843 265 [51.35%] male), 246 669 (15.02%) were born to mothers with a history of SA and 9750 (0.59%) were born to mothers with a history of stillbirth (Table 1). The SA-exposed group consisted of 206 174 offspring (12.55%) with a maternal history of 1 episode of SA, 33 534 (2.04%) with a maternal history of 2 episodes, and 6961 (0.42%) with a maternal history of 3 or more episodes. In the case of stillbirth, the exposed group consisted of 9455 (0.58%) offspring with a maternal history of single stillbirth and 295 (0.02%) with a maternal history of multiple stillbirths. As shown in Table 1, the prevalence of maternal history of SA and stillbirth increased with increasing birth order, maternal prepregnancy weight, and parental age at birth and those whose mothers were cohabitating at birth or were diagnosed with type 1 diabetes, type 2 diabetes, gestational diabetes, CHD, or hypothyroidism.

**Table 1.** Characteristics of Offspring With Proportions of Maternal History of SA and Stillbirth and Prevalence of Congenital Heart Disease^a^

Characteristic	All births	Births with maternal history of SA		Births with maternal history of stillbirth		Births with CHD	
		No. (%)	Prevalence, %	No. (%)	Prevalence, %	No. (%)	Prevalence, %
All	1 642 534 (100)	246 669 (100)	15.02	9750 (100)	0.59	30 318 (100)	1.85
Birth year							
1977-1991	283 433 (17.26)	43 664 (17.70)	15.41	1619 (16.61)	0.57	3052 (10.07)	1.08
1992-1996	304 387 (18.53)	50 236 (20.37)	16.50	2060 (21.13)	0.68	4542 (14.98)	1.49
1997-2001	308 449 (18.78)	51 775 (20.99)	16.79	1971 (20.22)	0.64	6119 (20.18)	1.98
2002-2006	301 545 (18.36)	48 720 (19.75)	16.16	1981 (20.32)	0.66	6208 (20.48)	2.06
2007-2011	256 893 (15.64)	35 553 (14.41)	13.84	1437 (14.74)	0.56	6200 (20.45)	2.41
2012-2016	187 827 (11.44)	16 721 (6.78)	8.90	682 (6.99)	0.36	4197 (13.84)	2.23
Sex							
Boys	843 265 (51.35)	126 250 (51.19)	14.97	4914 (50.41)	0.58	15 851 (52.28)	1.88
Girls	799 005 (48.65)	120 395 (48.81)	15.07	4835 (49.59)	0.61	14 467 (47.72)	1.81
Birth order							
1	793 391 (48.30)	75 374 (30.56)	9.50	330 (3.38)	0.04	14 952 (49.32)	1.88
2	589 646 (35.90)	103 035 (41.77)	17.47	3184 (32.66)	0.54	10 314 (34.02)	1.75
≥3	259 497 (15.80)	68 260 (27.67)	26.30	6236 (63.96)	2.40	5052 (16.66)	1.95
Maternal age at birth, y							
<20	47 364 (2.88)	2038 (0.83)	4.30	71 (0.73)	0.15	936 (3.09)	1.98
20-24	301 295 (18.34)	27 563 (11.17)	9.15	1063 (10.90)	0.35	5587 (18.43)	1.85
25-29	608 204 (37.03)	77 113 (31.26)	12.68	2972 (30.48)	0.49	11 013 (36.32)	1.81
30-34	487 158 (29.66)	87 342 (35.41)	17.93	3471 (35.60)	0.71	9036 (29.80)	1.85
≥35	198 513 (12.09)	52 613 (21.33)	26.50	2173 (22.29)	1.09	3746 (12.36)	1.89
Maternal education^b^							
Low	375 326 (23.47)	58 642 (24.13)	15.62	2685 (28.14)	0.72	7696 (25.89)	2.05
Medium	739 517 (46.23)	108 851 (44.79)	14.72	4378 (45.89)	0.59	13 622 (45.83)	1.84
High	484 647 (30.30)	75 540 (31.08)	15.59	2478 (25.97)	0.51	8404 (28.28)	1.73
Maternal cohabitation							
Single	813 725 (49.64)	102 677 (41.63)	12.62	3634 (37.28)	0.45	15 261 (50.38)	1.88
Cohabitation	825 594 (50.36)	143 980 (58.37)	17.44	6113 (62.72)	0.74	15 029 (49.62)	1.82
Maternal smoking							
No	1 157 761 (80.81)	178 495 (79.10)	15.42	6869 (77.88)	0.59	20 953 (78.92)	1.81
Yes	274 889 (19.19)	47 149 (20.90)	17.15	1951 (22.12)	0.71	5597 (21.08)	2.04
Maternal prepregnancy weight							
Underweight	31 990 (4.38)	4659 (3.93)	14.56	124 (2.71)	0.39	575 (4.29)	1.80
Normal weight	455 198 (62.29)	70 636 (59.56)	15.52	2318 (50.62)	0.51	7921 (59.13)	1.74
Overweight	153 375 (20.99)	26 831 (22.62)	17.49	1207 (26.36)	0.79	2972 (22.18)	1.94
Obesity	90 164 (12.34)	16 476 (13.89)	18.27	930 (20.31)	1.03	1929 (14.40)	2.14
Maternal prepregnancy type 1 diabetes							
No	1 636 617 (99.64)	245 419 (99.49)	15.00	9623 (98.70)	0.59	30 056 (99.14)	1.84
Yes	5917 (0.36)	1250 (0.51)	21.13	127 (1.30)	2.15	262 (0.86)	4.43
Maternal prepregnancy type 2 diabetes							
No	1 623 918 (98.87)	242 931 (98.48)	14.96	9474 (97.17)	0.58	29 728 (98.05)	1.83
Yes	18 616 (1.13)	3738 (1.52)	20.08	276 (2.83)	1.48	590 (1.95)	3.17
Maternal gestational diabetes							
No	1 622 803 (98.80)	242 853 (98.45)	14.97	9505 (97.49)	0.59	29 925 (98.70)	1.84
Yes	19 731 (1.20)	3816 (1.55)	19.34	245 (2.51)	1.24	393 (1.30)	1.99
Maternal CHD							
No	1 630 633 (99.28)	244 728 (99.21)	15.01	9596 (98.42)	0.59	29 507 (97.33)	1.81
Yes	11 901 (0.72)	1941 (0.79)	16.31	154 (1.58)	1.29	811 (2.67)	6.81
Maternal hypothyroidism							
No	1 602 825 (97.58)	239 774 (97.20)	14.96	9471 (97.14)	0.59	29 452 (97.14)	1.84
Yes	39 709 (2.42)	6895 (2.80)	17.36	279 (2.86)	0.70	866 (2.86)	2.18
Paternal age at birth, y							
<20	12 069 (0.75)	556 (0.23)	4.61	18 (0.19)	0.15	244 (0.82)	2.02
20-24	151 370 (9.38)	12 767 (5.24)	8.43	472 (4.90)	0.31	2827 (9.51)	1.87
25-29	472 483 (29.28)	54 505 (22.35)	11.54	2044 (21.24)	0.43	8683 (29.22)	1.84
30-34	550 013 (34.08)	86 372 (35.42)	15.70	3258 (33.86)	0.59	10 046 (33.80)	1.83
≥35	427 897 (26.51)	89 669 (36.77)	20.96	3831 (39.81)	0.90	7921 (26.65)	1.85

Abbreviations: CHD, congenital heart disease; SA, spontaneous abortion.

^a^ Values were missing for 264 participants for sex, 43 044 for maternal educational level, 3215 for maternal cohabitation, 209 884 for maternal smoking, 911 807 for maternal prepregnancy weight, and 28 702 for paternal age.

^b^ Low indicates primary and lower secondary education; medium, upper secondary education or academic degree; and high, university education at bachelor’s degree level or higher.

### Outcomes

The overall prevalence of CHD was 1.85% in offspring (30 318 children), with a corresponding prevalence of 1.88% in boys (15 851 children) and 1.81% in girls (14 467 children) (Table 1). The median time at diagnosis of CHD was 101 days after birth (IQR, birth to 3.15 years). In total, 18 273 cases (60.27%) were diagnosed before 1 year of age, 7064 (23.30%) were diagnosed between 1 and 4 years of age, 4658 (15.36%) were diagnosed between 5 and 17 years of age, and 323 (1.07%) were diagnosed in adulthood. The prevalence of CHD was much higher in offspring whose mothers had prepregnancy diabetes or CHD and was slightly higher in those with maternal smoking, prepregnancy overweight or obesity compared with normal weight, gestational diabetes, or hypothyroidism (Table 1). Most cases of prepregnancy type 2 diabetes were young-onset cases (median age of diagnosis, 27.15 [IQR, 23.35-30.31] years).

### Association of Risk of CHD With Maternal History of Pregnancy Loss

The adjusted HR of overall CHD was 1.16 (95% CI, 1.13-1.20) for offspring with a maternal history of SA and 1.49 (95% CI, 1.32-1.68) for those with a maternal history of stillbirth. Dose-response associations were observed for both SA and stillbirth. The HR increased from 1.12 (95% CI, 1.09-1.16) for 1 episode of SA to 1.29 (95% CI, 1.20-1.39) for 2 episodes of SA and 1.60 (95% CI, 1.39-1.84) for 3 or more episodes of SA. The risk of CHD for multiple stillbirths (HR, 2.75 [95% CI, 1.63-4.65]) was greater than that for single stillbirth (HR, 1.45 [95% CI, 1.29-1.64]) (Table 2). If only inpatient CHD cases were included, the risk of CHD was higher than that found in the main analysis, with an HR of 1.24 (95% CI, 1.19-1.30) for a maternal history of SA and an HR of 1.78 (95% CI, 1.51-2.11) for a maternal history of stillbirth, and similar dose-response patterns were observed (Table 2).

**Table 2.** Associations of Maternal History of SA or Stillbirth With CHD in Offspring

Exposure	No. of cases	No. of persons followed up	HR (95% CI)		*P* value for trend
			Crude	Adjusted^a^	
**Overall CHD**					
Maternal history of SA					
Unexposed	25 119	1 395 865	1 [Reference]	1 [Reference]	<.001
Exposed					
Any	5199	246 669	1.19 (1.15-1.22)	1.16 (1.13-1.20)	
1	4200	206 174	1.14 (1.11-1.18)	1.12 (1.09-1.16)	
2	794	33 534	1.34 (1.25-1.45)	1.29 (1.20-1.39)	
≥3	205	6961	1.70 (1.48-1.95)	1.60 (1.39-1.84)	
Maternal history of stillbirth					
Unexposed	30 042	1 632 784	1 [Reference]	1 [Reference]	<.001
Exposed					
Any	276	9750	1.56 (1.39-1.76)	1.49 (1.32-1.68)	
1	262	9455	1.52 (1.35-1.72)	1.45 (1.29-1.64)	
≥2	14	295	3.23 (1.91-5.45)	2.75 (1.63-4.65)	
**Inpatient CHD**					
Maternal history of SA					
Unexposed	11 221	1 382 100	1 [Reference]	1 [Reference]	<.001
Exposed					
Any	2359	243 848	1.20 (1.14-1.25)	1.24 (1.19-1.30)	
1	1899	203 890	1.15 (1.10-1.21)	1.20 (1.14-1.26)	
2	346	33 088	1.30 (1.16-1.44)	1.36 (1.22-1.51)	
≥3	114	6870	2.07 (1.72-2.49)	2.11 (1.76-2.55)	
Maternal history of stillbirth					
Unexposed	13 439	1 616 329	1 [Reference]	1 [Reference]	<.001
Exposed					
Any	141	9619	1.78 (1.51-2.10)	1.78 (1.51-2.11)	
1	230	9327	1.69 (1.42-2.0)	1.69 (1.42-2.02)	
≥2	11	292	4.87 (2.70-8.78)	4.52 (2.50-8.14)	

Abbreviations: CHD, congenital heart disease; HR, hazard ratio; SA, spontaneous abortion.

^a^ Adjusted for birth year, sex, birth order, maternal age at birth, maternal cohabitation, maternal educational level, maternal CHD, maternal hypothyroidism, and paternal age at birth.

### Joint Association of Maternal History of Pregnancy Loss and Maternal Diabetes With CHD

The estimates remained similar after adjustment for maternal diabetes, indicating the confounding effect of maternal diabetes was minimal (eTable 2 in the Supplement). We found that maternal prepregnancy type 2 diabetes modified the effect of maternal history of SA on CHD in offspring (*P* < .001 for interaction). The risk of CHD for offspring with a maternal history of SA and maternal prepregnancy type 2 diabetes increased by 177% (HR, 2.77; 95% CI, 2.38-3.21), compared with a 15% increase for those with only maternal history of SA (HR, 1.15; 95% CI, 1.11-1.18) and 63% increase for those with only maternal prepregnancy type 2 diabetes (HR, 1.63; 95% CI, 1.48-1.79). Correspondingly, a stronger association between maternal history of SA and CHD was observed in those with maternal prepregnancy type 2 diabetes than that in those without the disease (65% increase in CHD risk; HR, 1.65; 95% CI, 1.37-1.97) (Table 3). Although the number of children exposed to both a maternal history of stillbirth and maternal diabetes was small, we observed a similar but statistically nonsignificant pattern of prepregnancy type 2 diabetes strengthening the association of maternal history of stillbirth with CHD (74% increase; HR, 1.74; 95% CI, 1.06-2.85) (Table 3).

**Table 3.** Modification of Association of Maternal History of SA or Stillbirth With CHD in Offspring by Maternal Diabetes

Possible modifier	No maternal history of SA/stillbirth			Maternal history of SA/stillbirth			HR (95% CI) for maternal SA/stillbirth within strata of maternal diabetes^a^	*P* value for interaction
	No. of cases	No. of persons followed up	HR (95% CI)^a^	No. of cases	No. of persons followed up	HR (95% CI)^a^		
**Maternal history of SA**								
Prepregnancy type 1 diabetes								
No	24 919	1 391 198	1 [Reference]	5137	245 419	1.16 (1.12-1.19)	1.16 (1.12-1.19)	.89
Yes	200	4667	2.40 (2.09-2.76)	62	1250	2.72 (2.12-3.49)	1.12 (0.83-1.50)	
Prepregnancy type 2 diabetes								
No	24 705	1 380 987	1 [Reference]	5023	242 931	1.15 (1.11-1.18)	1.15 (1.11-1.18)	<.001
Yes	414	14 878	1.63 (1.48-1.79)	176	3738	2.77 (2.38-3.21)	1.65 (1.37-1.97)	
Gestational diabetes								
No	24 810	1 379 950	1 [Reference]	5115	242 853	1.16 (1.13-1.20)	1.16 (1.13-1.20)	.79
Yes	309	15 915	1.09 (0.97-1.22)	84	3816	1.22 (0.99-1.52)	1.19 (0.93-1.52)	
**Maternal history of stillbirth**								
Prepregnancy type 1 diabetes								
No	29 788	1 626 994	1 [Reference]	268	9623	1.47 (1.31-1.67)	1.47 (1.31-1.67)	.82
Yes	254	5790	2.39 (2.11-2.70)	8	127	3.23 (1.61-6.45)	1.36 (0.66-2.79)	
Prepregnancy type 2 diabetes								
No	29 469	1 614 444	1 [Reference]	259	9474	1.45 (1.28-1.64)	1.45 (1.28-1.64)	.24
Yes	573	18 340	1.79 (1.65-1.95)	17	276	3.48 (2.17-5.61)	1.74 (1.06-2.85)	
Gestational diabetes								
No	29 654	1 613 298	1 [Reference]	271	9505	1.50 (1.33-1.70)	1.50 (1.33-1.70)	.33
Yes	388	19 486	1.09 (0.99-1.20)	5	245	1.05 (0.44-2.51)	1.14 (0.47-2.78)	

Abbreviations: CHD, congenital heart disease; HR, hazard ratio; SA, spontaneous abortion.

^a^ Adjusted for birth year, sex, birth order, maternal age at birth, maternal cohabitation, maternal educational level, maternal CHD, maternal hypothyroidism, and paternal age at birth.

### Subgroup Analyses and Sensitivity Analyses

Dealing with missing values of covariates by multiple imputation did not substantially change the result, compared with that obtained after excluding participants with missing values of covariates (eTable 3 in the Supplement). The risk of CHD did not differ by sex of offspring (eTable 4 in the Supplement). We found similar results when we restricted the analyses to first live-born children (eTable 5 in the Supplement), to offspring born after 1995 (eTables 6 and 7 in the Supplement), and to offspring born after 1994 (eTable 8 in the Supplement). We observed similar HRs after we redefined SA and stillbirth using 28 gestational weeks as the cutoff point for diagnosis (eTable 9 in the Supplement). Additional adjustment for maternal smoking or maternal prepregnancy body mass index did not essentially change the overall association (eTables 10 and 11 in the Supplement).

## Discussion

In this population-based cohort study of offspring born between 1977 and 2016 in Denmark, we found that maternal history of SA and stillbirth were associated with increased risks of CHD in offspring of 16% and 49%, respectively. Dose-response associations were found with a higher risk of CHD after multiple episodes of maternal SA or stillbirth. The association was further strengthened by maternal prepregnancy type 2 diabetes, with the risks of CHD increased among those with maternal history of SA and stillbirth by 65% and 74%, respectively.

Our findings that maternal history of SA and stillbirth were associated with higher risk of CHD are in line with those of most previous studies.^10,11,12^ A Swedish case-control study found a 22% increased risk of CHD associated with a maternal history of SA and an 89% increased risk of CHD associated with a maternal history of perinatal death.^10^ Another case-control study in the US^11^ also reported dose-response associations—namely, a 29% increased risk of CHD associated with maternal history of 1 SA and a 62% increased risk associated with 2 SAs. A hospital-based study in Southern Israel^12^ found 36% increased risk of CHD after maternal recurrent SA (≥3 SAs).

We also found that prepregnancy type 2 diabetes strengthened the effects of maternal history of SA and stillbirth on CHD. Type 2 diabetes is characterized by relative insulin deficiency caused by pancreatic β-cell dysfunction and insulin resistance in target organs. The incretin effect, immune dysregulation, and inflammation have emerged as important pathophysiological factors of type 2 diabetes.^20^ In particular, young-onset (15-30 years of age) type 2 diabetes appears to have more profound complications than type 1 diabetes diagnosed in people at a similar age and with equivalent diabetes duration.^21^ In case of pregnancy loss, immune abnormalities and inflammatory responses play a key role in its pathophysiology.^22^ Most cases of prepregnancy type 2 diabetes in our study were young-onset cases, which may be an indication of more severe pathophysiological factors. Therefore, prepregnancy type 2 diabetes and SA or stillbirth in women of reproductive age could share common pathways in pathogenesis (such as immune or inflammatory abnormalities), thus contributing jointly to CHD in offspring. Nevertheless, further studies are warranted to clarify the joint association between prepregnancy type 2 diabetes and pregnancy loss.

The underlying mechanisms linking maternal history of SA and stillbirth to the development of CHD are yet to be elucidated. Evidence shows that the proportion of CHD among spontaneous abortuses and stillborn fetuses is much higher than that among live-born infants.^23^ Owing to the strong familial aggregation of discordant CHD phenotypes,^24^ it is plausible that the risk of CHD in live-born children after a maternal history of SA or stillbirth is higher. Recent studies have suggested that genetic variants related to the synthesis pathway of nicotinamide adenine dinucleotide, a ubiquitous biological molecule that participates in many metabolic reactions, cause congenital malformation and SA in humans and mice,^25,26^ indicating that SA and CHD may share common genetic abnormalities. Furthermore, previous SA may be related to defects of the placenta,^27^ and placental abnormalities may involve the development of CHD.^28^ These findings support the biological plausibility of our observed association, though further research is needed.

### Strengths and Limitations

The large sample size in this study enabled us to examine the association of maternal history of maternal SA or stillbirth with CHD in offspring and to perform subgroup analyses to further verify the associations. We were also able to adjust for a wide range of covariates using the high-quality registry data to obtain more reliable estimates. In addition, our cohort study included nearly all eligible study participants in Denmark with almost complete follow-up data, which minimized the potential influence of selection bias.

Our findings should also be interpreted within the context of the following limitations. First, misclassification of SA may exist, because the validation study for the diagnosis of SA in the DNPR showed that approximately 30% of SAs reported by women were not registered in the DNPR.^29^ However, only 3% of SA diagnoses in the DNPR did not fulfill the criteria of SA, suggesting that registration of SA in DNPR accurately reflected the diagnoses recorded in medical records.^30^ Although we could not rule out potential misclassification bias, such bias would likely be nondifferential and would tend to attenuate our results. Therefore, potential misclassification bias is unlikely to change our conclusion substantially. Second, although several validation studies have suggested that the DNPR is a valid resource for epidemiological studies looking at CHD as a group,^31,32,33^ errors in hospital records cannot be avoided owing to the long study period and the coverage of all hospitals in Denmark. Studies based on individual review are warranted to verify our findings. Third, we did not test the joint associations of other maternal diseases, such as hypothyroidism and obesity. Future studies are needed to corroborate the potential joint associations of other maternal diseases. Fourth, although we adjusted for a wide range of potential confounders, we could not rule out the potential residual effects from unmeasured or undefined confounders. Fifth, we did not consider nonregistered pregnancy loss, and generalization of our findings should be cautious in case of pregnancy loss not requiring a hospital contact.

## Conclusions

The findings of this cohort study contribute to a body of evidence that children of mothers who have a history of SA or stillbirth are at a higher risk of being diagnosed with CHD. The risk increased with more episodes of SA or stillbirth experienced by the mothers. In addition, children had a much higher risk of CHD if their mothers had prepregnancy type 2 diabetes concurrently. These findings may help to identify high-risk groups for detailed, cost-effective fetal heart assessment that facilitates prenatal diagnosis or early diagnosis after birth to gain improved perioperative outcomes for children with certain forms of CHD. Our findings also highlight the importance of screening for type 2 diabetes in women of reproductive age.

## Notes

Supplement. **eTable 1.** *ICD* Codes Used for Diagnoses **eTable 2.** Comparison Between the Crude Estimates and Estimates Adjusted for Maternal Diabetes in Associations of Maternal History of SA and Stillbirth With CHD in Offspring **eTable 3.** Associations of Maternal History of SA and Stillbirth With CHD in Offspring **eTable 4.** Associations of Maternal History of SA and Stillbirth With CHD in Offspring by Sex of Offspring **eTable 5.** Associations of Maternal History of SA and Stillbirth With CHD Among First Live-born Children (n = 825 847) **eTable 6.** Associations of Maternal History of SA and Stillbirth With CHD in Among Offspring Born After 1995 (n = 1 309 773) **eTable 7.** Modification of Association of Maternal History of SA or Stillbirth With CHD by Maternal Diabetes Among Offspring Born After 1995 (n = 1 309 773) **eTable 8.** Associations of Maternal History of SA and Stillbirth With CHD Among Offspring Born After 1994 (n = 1 362 413) **eTable 9.** Associations of Maternal History of SA and Stillbirth With CHD in Offspring, Redefining SA and Stillbirth by 28 Gestational Weeks **eTable 10.** Associations Between Maternal History of SA and Stillbirth and CHD Additionally Adjusting for Maternal Smoking Among Offspring Born After 1991 (n = 1 493 423) **eTable 11.** Associations of Maternal History of SA and Stillbirth With CHD Additionally Adjusting for Maternal Prepregnancy BMI Among Offspring Born After 2004 (n = 774 025)
