# Supplementary material for: Association of Maternal History of Spontaneous Abortion and Stillbirth With Risk of Congenital Heart Disease in Offspring of Women With vs Without Type 2 Diabetes
Source: JAMA Netw Open. 2021 Nov 10;4(11):e2133805. doi: 10.1001/jamanetworkopen.2021.33805 (PMC8581719; doi:10.1001/jamanetworkopen.2021.33805)
Supplement: Supplement. — eTable 1. ICD Codes Used for Diagnoses eTable 2. Comparison Between the Crude Estimates and Estimates Adjusted for Maternal Diabetes in Associations of Maternal History of SA and Stillbirth With CHD in Offspring eTable 3. Associations of Maternal History of SA and Stillbirth With CHD in Offspring eTable 4. Associations of Maternal History of SA and Stillbirth With CHD in Offspring by Sex of Offspring eTable 5. Associations of Maternal History of SA and Stillbirth With CHD Among First Live-born Children (n = 825 847) eTable 6. Associations of Maternal History of SA and Stillbirth With CHD in Among Offspring Born After 1995 (n = 1 309 773) eTable 7. Modification of Association of Maternal History of SA or Stillbirth With CHD by Maternal Diabetes Among Offspring Born After 1995 (n = 1 309 773) eTable 8. Associations of Maternal History of SA and Stillbirth With CHD Among Offspring Born After 1994 (n = 1 362 413) eTable 9. Associations of Maternal History of SA and Stillbirth With CHD in Offspring, Redefining SA and Stillbirth by 28 Gestational Weeks eTable 10. Associations Between Maternal History of SA and Stillbirth and CHD Additionally Adjusting for Maternal Smoking Among Offspring Born After 1991 (n = 1 493 423) eTable 11. Associations of Maternal History of SA and Stillbirth With CHD Additionally Adjusting for Maternal Prepregnancy BMI Among Offspring Born After 2004 (n = 774 025) [file jamanetwopen-e2133805-s001.pdf]

Supplementary Online Content

Ji H, Liang H, Yu Y, et al. Association of maternal history of spontaneous abortion and stillbirth with risk of congenital heart disease in offspring of women with vs without type 2 diabetes. *JAMA Netw Open*. 2021;4(11):e2133805.
doi:10.1001/jamanetworkopen.2021.33805

eTable 1. *ICD* Codes Used for Diagnoses
eTable 2. Comparison Between the Crude Estimates and Estimates Adjusted for Maternal Diabetes in Associations of Maternal History of SA and Stillbirth With CHD in Offspring
eTable 3. Associations of Maternal History of SA and Stillbirth With CHD in Offspring
eTable 4. Associations of Maternal History of SA and Stillbirth With CHD in Offspring by Sex of Offspring
eTable 5. Associations of Maternal History of SA and Stillbirth With CHD Among First Live-born Children (n = 825 847)
eTable 6. Associations of Maternal History of SA and Stillbirth With CHD in Among Offspring Born After 1995 (n = 1 309 773)
eTable 7. Modification of Association of Maternal History of SA or Stillbirth With CHD by Maternal Diabetes Among Offspring Born After 1995 (n = 1 309 773)
eTable 8. Associations of Maternal History of SA and Stillbirth With CHD Among Offspring Born After 1994 (n = 1 362 413)
eTable 9. Associations of Maternal History of SA and Stillbirth With CHD in Offspring, Redefining SA and Stillbirth by 28 Gestational Weeks
eTable 10. Associations Between Maternal History of SA and Stillbirth and CHD Additionally Adjusting for Maternal Smoking Among Offspring Born After 1991 (n = 1 493 423)
eTable 11. Associations of Maternal History of SA and Stillbirth With CHD Additionally Adjusting for Maternal Prepregnancy BMI Among Offspring Born After 2004 (n = 774 025)

This supplementary material has been provided by the authors to give readers additional information about their work.

© 2021 Ji H et al. *JAMA Network Open*.

eTable 1. *ICD* Codes Used for Diagnoses

Diagnosis	ICD-8	ICD-10
Spontaneous abortion	6346, 6451, 6430, 6438, 6439	O021, O03
Congenital heart disease	746-747<br>(except for 746.7 and 747.5-747.9)	Q20-Q26<br>(except for Q26.5-Q26.6)
Type 1 diabetes	249	E10, O24.0
Type 2 diabetes	250	E11, O24.1
Gestational diabetes	634.74, Y6449	O24.4, O24.9
Hypothyroidism	243, 244	E03, E890

© 2021 Ji H et al. *JAMA Network Open*.

eTable 2. Comparison Between the Crude Estimates and Estimates Adjusted for Maternal Diabetes in Associations of Maternal History of SA and Stillbirth With CHD in Offspring

Exposure	Cases	Followed Persons	HR (95% CI)			
			Crude	Adjusted [a]	Adjusted [b]	Adjusted [c]
**Maternal history of SA**						
Unexposed	25 119	1 395 865	ref	ref	ref	ref
Exposed	5 199	246 669	1.19 (1.15, 1.22)	1.19 (1.15, 1.22)	1.18 (1.15, 1.22)	1.19 (1.15, 1.22)
1	4 200	206 174	1.14 (1.11, 1.18)	1.14 (1.11, 1.18)	1.14 (1.11, 1.18)	1.14 (1.11, 1.18)
2	794	33 534	1.34 (1.25, 1.45)	1.34 (1.25, 1.44)	1.33 (1.24, 1.43)	1.34 (1.25, 1.44)
≥3	205	6 961	1.70 (1.48, 1.95)	1.70 (1.48, 1.95)	1.69 (1.47, 1.94)	1.71 (1.49, 1.96)
**Maternal history of stillbirth**						
Unexposed	30 042	1 632 784	ref	ref	ref	ref
Exposed	276	9 750	1.56 (1.39, 1.76)	1.52 (1.35, 1.72)	1.52 (1.35, 1.72)	1.54 (1.37, 1.74)
1	262	9 455	1.52 (1.35, 1.72)	1.48 (1.31, 1.68)	1.48 (1.31, 1.67)	1.50 (1.33, 1.70)
≥2	14	295	3.23 (1.91, 5.45)	3.08 (1.83, 5.20)	3.18 (1.89, 5.37)	3.24 (1.92, 5.46)

SA, spontaneous abortion.

Cox proportional hazard regression.

[a] Adjusted for maternal prepregnancy type 1 diabetes.

[b] Adjusted for maternal prepregnancy type 2 diabetes.

[c] Adjusted for maternal gestational diabetes.

© 2021 Ji H et al. *JAMA Network Open*.

eTable 3. Associations of Maternal History of SA and Stillbirth With CHD in Offspring

Exposure	Cases	Followed Persons	HR (95% CI)	
			Adjusted [a]	Adjusted [b]
**Maternal history of SA**				
Unexposed	25 119	1 395 865	ref	ref
Exposed	5 199	246 669	1.16 (1.13, 1.20)	1.15 (1.12, 1.19)
1	4 200	206 174	1.12 (1.09, 1.16)	1.12 (1.08, 1.15)
2	794	33 534	1.29 (1.20, 1.39)	1.29 (1.20, 1.39)
≥3	205	6 961	1.60 (1.39, 1.84)	1.58 (1.37, 1.81)
**Maternal history of stillbirth**				
Unexposed	30 042	1 632 784	ref	ref
Exposed	276	9 750	1.49 (1.32, 1.68)	1.48 (1.31, 1.67)

SA, spontaneous abortion.

Cox proportional hazard regression.

Adjusted for birth year, sex, birth order, maternal age at birth, maternal cohabitation, maternal education, maternal congenital heart disease, maternal hypothyroidism and paternal age at birth.

[a] Main result: using multiple imputation to deal with missing values covariates including child sex, maternal cohabitation, maternal education, and paternal age at birth.

[b] Excluding subjects with missing values of covariates.

© 2021 Ji H et al. *JAMA Network Open*.

eTable 4. Associations of Maternal History of SA and Stillbirth With CHD in Offspring by Sex of Offspring

Exposure	Cases	Followed persons	HR (95% CI)	
			Crude	Adjusted
Boys (N=843 265)				
**Maternal history of SA**				
Unexposed	13 172	717 015	ref	ref
Exposed	2 679	126 250	1.17 (1.12, 1.22)	1.14 (1.09, 1.19)
1	2 197	105 668	1.14 (1.09, 1.20)	1.12 (1.07, 1.17)
2	383	17 082	1.24 (1.12, 1.37)	1.19 (1.08, 1.32)
≥3	99	3 500	1.60 (1.31, 1.95)	1.49 (1.22, 1.82)
**Maternal prior stillbirth**				
Unexposed	15 704	838 351	ref	ref
Exposed	147	4 914	1.62 (1.37, 1.90)	1.53 (1.30, 1.80)
Girls (N=799 005)				
**Maternal history of SA**				
Unexposed	11 947	678 610	ref	ref
Exposed	2 520	120 395	1.21 (1.16, 1.26)	1.18 (1.13, 1.23)
1	2 003	100 488	1.15 (1.09, 1.20)	1.13 (1.08, 1.18)
2	411	16 446	1.45 (1.31, 1.60)	1.40 (1.27, 1.55)
≥3	106	3 461	1.82 (1.50, 2.20)	1.72 (1.42, 2.08)
**Maternal history of stillbirth**				
Unexposed	14 338	794 170	ref	ref
Exposed	129	4 835	1.50 (1.26, 1.79)	1.45 (1.21, 1.72)

SA, spontaneous abortion.

Cox proportional hazard regression.

Adjusted for birth year, birth order, maternal age at birth, maternal cohabitation, maternal education, maternal congenital heart disease, maternal hypothyroidism and paternal age at birth.

© 2021 Ji H et al. *JAMA Network Open*.

eTable 5. Associations of Maternal History of SA and Stillbirth With CHD Among First Live-born Children (n = 825 847)

Exposure	Cases	Followed persons	HR (95% CI)	
			Crude	Adjusted
**Maternal history of SA**				
Unexposed	13 786	746 992	ref	ref
Exposed	1 731	78 855	1.20 (1.14, 1.26)	1.16 (1.10, 1.22)
1	1 490	69 536	1.17 (1.11, 1.23)	1.14 (1.08, 1.20)
2	199	8 119	1.34 (1.17, 1.54)	1.28 (1.11, 1.47)
≥3	42	1 200	1.95 (1.44, 2.64)	1.79 (1.32, 2.43)
**Maternal history of stillbirth**				
Unexposed	15 419	822 385	ref	ref
Exposed	98	3 462	1.52 (1.25, 1.86)	1.66 (1.33, 2.07)

SA, spontaneous abortion.

Cox proportional hazard regression.

Adjusted for birth year, sex, birth order, maternal age at birth, maternal cohabitation, maternal education, maternal congenital heart disease, maternal hypothyroidism and paternal age at birth.

© 2021 Ji H et al. *JAMA Network Open*.

eTable 6. Associations of Maternal History of SA and Stillbirth With CHD in Among Offspring Born After 1995 (n = 1 309 773)

Exposure	Cases	Followed persons	HR (95% CI)	
			Crude	Adjusted
**Maternal history of SA**				
Unexposed	20 269	1 098 564	ref	ref
Exposed	4 497	211 209	1.15 (1.11, 1.19)	1.15 (1.12, 1.19)
1	3 619	175 017	1.12 (1.08, 1.16)	1.12 (1.08, 1.16)
2	693	29 799	1.26 (1.16, 1.35)	1.26 (1.16, 1.36)
≥3	185	6 393	1.59 (1.37, 1.84)	1.56 (1.35, 1.81)
**Maternal history of stillbirth**				
Unexposed	24 525	1 301 484	ref	ref
Exposed	241	8 289	1.55 (1.36, 1.76)	1.52 (1.33, 1.72)

SA, spontaneous abortion.

Cox proportional hazard regression.

Adjusted for birth year, sex, birth order, maternal age at birth, maternal cohabitation, maternal education, maternal congenital heart disease, maternal hypothyroidism and paternal age at birth.

© 2021 Ji H et al. *JAMA Network Open*.

eTable 7. Modification of Association of Maternal History of SA or Stillbirth With CHD by Maternal Diabetes Among Offspring Born After 1995 (n = 1 309 773)

Possible modifier	No maternal history of SA/stillbirth			Maternal history of SA/stillbirth			HR (95% CI) for maternal SA/stillbirth within strata of maternal diabetes	P for interaction
	Cases	Followed persons	HR (95% CI)	Cases	Followed persons	HR (95% CI)		
Maternal history of SA								
**Prepregnancy type 1 diabetes**								
No	20 000	1 094 083	ref	4 425	209 995	1.15 (1.12, 1.19)	1.15 (1.12, 1.19)	0.90
Yes	187	4 481	2.33 (2.01, 2.69)	58	1 214	2.63 (2.04, 3.41)	1.09 (0.80, 1.48)	
**Prepregnancy type 2 diabetes**								
No	19 817	1 084 715	ref	4 326	207 670	1.14 (1.10, 1.18)	1.14 (1.10, 1.18)	<0.001
Yes	370	13 849	1.56 (1.40, 1.73)	157	3 539	2.60 (2.22, 3.05)	1.55 (1.28, 1.88)	
**Gestational diabetes**								
No	19 890	1 083 218	ref	4 401	207 491	1.15 (1.12, 1.19)	1.15 (1.12, 1.19)	0.96
Yes	297	15 346	1.26 (1.14, 1.38)	82	3 718	1.46 (1.21, 1.74)	1.19 (0.96, 1.46)	
Maternal history of stillbirth								
**Prepregnancy type 1 diabetes**								
No	24 193	1 295 911	ref	232	8 167	1.50 (1.32, 1.71)	1.50 (1.32, 1.71)	0.68
Yes	238	5 573	2.32 (2.04, 2.64)	7	122	2.95 (1.41, 6.19)	1.28 (0.59, 2.76)	
**Prepregnancy type 2 diabetes**								
No	23 921	1 284 351	ref	222	8 034	1.46 (1.27, 1.66)	1.46 (1.27, 1.66)	0.10

© 2021 Ji H et al. *JAMA Network Open*.

Yes	510	17 133	1.70 (1.56, 1.86)	17	255	3.78 (2.35, 6.08)	1.88 (1.15,3.08)	
**Gestational diabetes**								
No	24 056	1 282 653	ref	235	8 056	1.54 (1.36, 1.76)	1.54 (1.36, 1.76)	0.07
Yes	375	18 831	1.27 (1.17, 1.38)	4	233	0.85 (0.36, 2.05)	0.74 (0.31, 1.78)	

SA, spontaneous abortion.

Cox proportional hazard regression.

Adjusted for birth year, sex, birth order, maternal age at birth, maternal cohabitation, maternal education, maternal congenital heart disease, maternal hypothyroidism and paternal age at birth.

© 2021 Ji H et al. *JAMA Network Open*.

eTable 8. Associations of Maternal History of SA and Stillbirth With CHD Among Offspring Born After 1994 (n = 1 362 413)

Exposure	Cases	Followed persons	HR (95% CI)	
			Crude	Adjusted
**Maternal history of SA**				
Unexposed	20 902	1 144 124	ref	ref
Exposed	4 599	218 289	1.15 (1.11, 1.19)	1.15 (1.12, 1.19)
1	3 703	181 067	1.12 (1.08, 1.16)	1.12 (1.08, 1.16)
2	705	30 681	1.26 (1.17, 1.35)	1.25 (1.16, 1.35)
≥3	191	6 541	1.62 (1.41, 1.87)	1.59 (1.38, 1.83)
**Maternal history of stillbirth**				
Unexposed	25 258	1 353 805	ref	ref
Exposed	243	8 608	1.52 (1.34, 1.72)	1.49 (1.31, 1.69)

SA, spontaneous abortion.

Cox proportional hazard regression.

Adjusted for birth year, sex, birth order, maternal age at birth, maternal cohabitation, maternal education, maternal congenital heart disease, maternal hypothyroidism and paternal age at birth.

© 2021 Ji H et al. *JAMA Network Open*.

eTable 9. Associations of Maternal History of SA and Stillbirth With CHD in Offspring, Redefining SA and Stillbirth by 28 Gestational Weeks

Exposure	Cases	Followed persons	HR (95% CI)	
			Crude	Adjusted
**Maternal history of SA**				
Unexposed	25 098	1 395 189	Ref	ref
Exposed	5 220	247 345	1.19 (1.15, 1.22)	1.16 (1.13, 1.20)
1	4 210	206 558	1.15 (1.11, 1.18)	1.13 (1.09, 1.16)
2	803	33 739	1.35 (1.26, 1.45)	1.30 (1.21, 1.40)
≥3	207	7 048	1.70 (1.48, 1.95)	1.60 (1.39, 1.83)
**Maternal history of stillbirth**				
Unexposed	30 076	1 633 863	Ref	ref
Exposed	242	8 671	1.51 (1.33, 1.71)	1.46 (1.29, 1.66)

SA, spontaneous abortion.

Cox proportional hazard regression.

Adjusted for birth year, sex, birth order, maternal age at birth, maternal cohabitation, maternal education, maternal congenital heart disease, maternal hypothyroidism and paternal age at birth.

© 2021 Ji H et al. *JAMA Network Open*.

eTable 10. Associations Between Maternal History of SA and Stillbirth and CHD Additionally Adjusting for Maternal Smoking Among Offspring Born After 1991 (n = 1 493 423)

Exposure	Cases	Followed persons	HR (95% CI)	
			Adjusted [a]	Adjusted [b]
**Maternal history of SA**				
Unexposed	22 957	1 259 090	Ref	ref
Exposed	4 941	234 333	1.15 (1.12, 1.19)	1.15 (1.12, 1.19)
1	3 984	194 999	1.12 (1.08, 1.16)	1.12 (1.08, 1.16)
2	758	32 511	1.27 (1.18, 1.37)	1.27 (1.18, 1.36)
≥3	199	6 823	1.58 (1.37, 1.82)	1.58 (1.37, 1.81)
**Maternal history of stillbirth**				
Unexposed	27 638	1 484 202	Ref	ref
Exposed	260	9 221	1.48 (1.31, 1.68)	1.48 (1.31, 1.68)

SA, spontaneous abortion.

Cox proportional hazard regression.

[a] Adjusted for birth year, sex, birth order, maternal age at birth, maternal cohabitation, maternal education, maternal congenital heart disease, maternal hypothyroidism and paternal age at birth.

[b] additionally adjusted for maternal smoking.

© 2021 Ji H et al. *JAMA Network Open*.

eTable 11. Associations of Maternal History of SA and Stillbirth With CHD Additionally Adjusting for Maternal Prepregnancy BMI Among Offspring Born After 2004 (n = 774 025)

Exposure	Cases	Followed persons	HR (95% CI)	
			Adjusted [a]	Adjusted [b]
**Maternal history of SA**				
Unexposed	11 576	648 765	ref	ref
Exposed	2 586	125 260	1.16 (1.08, 1.21)	1.15 (1.10, 1.21)
1	2 051	103 470	1.11 (1.06, 1.17)	1.11 (1.06, 1.17)
2	421	17 822	1.32 (1.19, 1.45)	1.31 (1.19, 1.45)
≥3	114	3 968	1.60 (1.33, 1.92)	1.59 (1.32, 1.91)
**Maternal history of stillbirth**				
Unexposed	14 028	769 186	ref	ref
Exposed	134	4 839	1.48 (1.24, 1.75)	1.46 (1.23, 1.73)

SA, spontaneous abortion.

Cox proportional hazard regression.

[a] Adjusted for birth year, sex, birth order, maternal age at birth, maternal cohabitation, maternal education, maternal congenital heart disease, maternal hypothyroidism and paternal age at birth.

[b] additionally adjusted for maternal prepregnancy BMI.

© 2021 Ji H et al. *JAMA Network Open*.
